# What does satisfaction with wait times mean to cancer patients?

**DOI:** 10.1186/s12885-015-2041-z

**Published:** 2015-12-28

**Authors:** Maria Mathews, Dana Ryan, Donna Bulman

**Affiliations:** 1 Division of Community Health & Humanities, Health Sciences Centre, Room 2837, St. John’s, NL A1B 3V6 Canada; 2 Division of Community Health & Humanities, Health Sciences Centre, Room 2849, St. John’s, NL A1B 3V6 Canada; 3 Faculty of Nursing, University of New Brunswick, Fredericton, NB E3B 5A3 Canada

**Keywords:** Cancer care, Satisfaction, Wait times, Patient experiences, Patient-centered care

## Abstract

**Background:**

Patient satisfaction is an important element of quality improvement and patient-centered care, and is an indicator of the public’s confidence in the health care system. Although shorter wait times are believed intuitively to lead to higher satisfaction, studies have demonstrated the importance of many other factors which contribute to patients’ satisfaction with their wait time experiences. The current study explores the factors that shape patients’ satisfaction with their overall wait times (i.e. from symptom to treatment).

**Methods:**

We conducted qualitative interviews with 60 breast, prostate, lung, or colorectal cancer patients to examine the reasons behind patients’ satisfaction or dissatisfaction with their wait time experiences. We purposefully recruited satisfied and unsatisfied participants from our larger survey sample. Using a semi-structured interview guide, patients were asked about their wait time experiences and the reasons behind their (dis)satisfaction. Interviews were transcribed verbatim and coded using a thematic approach.

**Results:**

Patients’ perceptions of satisfaction with wait times were influenced by three interrelated dimensions: the interpersonal skills of treating physicians (which included expressions/demonstrations of empathy and concern, quality of information exchange, accountability for errors), coordination (which included assistance navigating the health system, scheduling of appointments, sharing information between providers, coordination in scheduling appointments, and sharing of information ), and timeliness of care (which referred to providers’ responsiveness to patients’ symptoms, coverage during provider absences, and shared sense of urgency between patient and providers). Providers’ willingness to “trouble shoot” and acknowledge errors/delays were particularly influential in patients' overall perception of their wait times.

**Conclusions:**

We described three dimensions of wait-related satisfaction: physicians’ interpersonal skills, coordination of care, and timeliness of care, which are often interrelated and overlapping. Furthermore, while patients wait-related satisfaction was typically based on multiple interactions with different providers, positive or negative experiences with a single provider, often (but not always) the family physician, had a substantial impact on the overall satisfaction or dissatisfaction with wait time experiences. The findings provide a conceptual basis for the development of validated instruments to measure wait time-related patient satisfaction.

## Background

Improving wait times for diagnosis and treatment for cancer care has been a priority in many jurisdictions. In addition to the clinical consequences of lengthy delays (e.g. increasing disease severity and disease-related morbidity [1]), public perception of wait times serve as a barometer of the public’s confidence in the health care system [2]. Studies suggest that the relationship between wait times and wait-related satisfaction is neither linear nor straightforward [3–6]; although shorter wait times are believed intuitively to lead to better wait related-satisfaction, many elements contribute to patients’ satisfaction with their wait time experiences. A study of colorectal cancer patients showed a moderate correlation between shorter wait times and higher levels of satisfaction [3]. Surveys of cancer patients found that the level of wait-related satisfaction varied by patient and treatment-related factors, such as type of cancer, treatment, prognosis, and personal screening behaviors [4, 5]. Moreover, the nature of the delay, whether it was avoidable or unavoidable or attributable to the patient, provider, or health system does not explain wait-related satisfaction or dissatisfaction [6].

Given the counter-intuitive findings between length of wait and satisfaction, a deeper understanding of what satisfaction with wait times means to patients is needed. In this study, we used qualitative interviews to explore the factors that shape patients’ satisfaction with overall wait times (i.e. from symptom to treatment). Our study examines waits from a holistic, patient-centered perspective as they move across sectors of the health system. Similar to measures of timeliness of care, patient satisfaction is recognized as an important element of quality improvement and patient-centred care [7–9]. The study sheds light on what patients value in relation to wait times management and helps to provide greater context to survey and polling data. The study is part of a larger project examining wait times in cancer care.

## Methods

This study was approved by the Memorial University Human Investigation Committee. Participants were selected from a pool of cancer patients who had been recruited to participate in a survey as an earlier part of a larger study [4]. All participants had received care for a cancer diagnosis at regional cancer clinics in Newfoundland and Labrador (NL). To be eligible for the study, participants needed to be 19 years of age or older, English speaking, residents of NL, and diagnosed with either breast, lung, colorectal, or prostate cancer. Patients with previous or multiple cancer diagnoses were excluded to control for possible influences on wait times. Prior to their participation, patients were required to sign a written consent form that explained the details of the study.

For this study, we used purposive sampling [10, 11], and invited participants to take part in a qualitative interview regarding their experiences with cancer care and wait times. From data gathered during the initial survey, participants were chosen based on their type of cancer (breast, lung, colorectal, or prostate), community of residence (urban- population ≥ 100,000; semi-urban- population 10,000 to 99,999; rural- population < 10,000), and level of satisfaction with their waiting times for cancer care (satisfied or unsatisfied with any wait time interval). A minimum of three people were interviewed to represent each type of community and cancer type, and both satisfied and unsatisfied patients were included among each group in the sample. Interviews continued until saturation of data was reached.

Interviews were conducted either in-person or by phone and ranged between eight and 82 min in length. During the interviews, participants were asked semi-structured questions regarding their wait times and perceptions of care received from the onset of symptoms to getting treatment for their cancer. They were also asked what improvements could have been made to expedite their wait times, what barriers, if any, they experienced when trying to access timely care, and how satisfied or unsatisfied they were with their wait times overall. Questions were open-ended, allowing patients the opportunity to talk at length about any particular issues they believed were relevant or influential to their wait time-related experiences.

Following collection of the data, interviews were transcribed verbatim and a thematic analysis was conducted [10, 12]. A number of steps were taken to ensure the accuracy of the analysis. For example, each interview transcript was compared against the digital recording in order to ensure it was correctly transcribed. Following this, each member of the research team read all transcripts in order to identify key themes and concepts. The identification and classification of these themes was routinely discussed amongst the research team and compared across interviews [11]. A coding template was then developed [10, 12] and routinely adjusted and expanded during analysis in an effort to ensure that forcing of data into categories did not occur. The final template was then used to code all interviews. An audit trail consisting of all transcripts, notes, and coding templates were entered into the system and coded using NVivo 9 software, allowing us to track analytic decisions and their rationales. To protect patient confidentiality, all participants were assigned a study ID number (which appear in brackets {} after each quotation). All quotations have been edited to ensure that any identifying information has been removed.

## Results

Of the 128 people invited to participate, 60 participants completed the interview. Participants’ demographic characteristics and satisfaction from first visit with a health care professional to diagnosis are presented in Table 1.

**Table 1:** Characteristics of interview participants by cancer type

	Breast	Colorectal	Lung	Prostate	All types
Characteristics	(*n* = 18)	(*n* = 15)	(*n* = 11)	(*n* = 16)	(*n* = 60)
Sex n (%)					
Male	0 (0)	11 (73.3)	6 (54.5)	16 (100.0)	33 (55.0)
Female	18 (100)	4 (26.7)	5 (45.5)	0 (0)	27 (45.0)
Age n (%)					
Under 65	16 (88.9)	10 (66.7)	6 (54.5)	8 (50.0)	40 (66.7)
65 and over	2 (11.1)	5 (33.3)	5 (45.5)	8 (50.0)	20 (33.3)
Community of Residence n (%)					
Urban	6 (33.3)	5 (33.3)	1 (9.1)	3 (18.8)	15 (25.0)
Semi-urban	6 (33.3)	4 (26.7)	2 (18.2)	4 (25.0)	16 (26.7)
Rural	6 (33.3)	6 (40.0)	8 (72.7)	9 (56.3)	29 (48.3)
Marital Status n (%)					
Married or Equivalent	16 (88.9)	13 (86.7)	10 (90.9)	16 (100.0)	55 (91.7)
Single	2 (11.1)	2 (13.3)	1 (9.1)	0 (0)	5 (8.3)
Satisfaction: 1st Visit - Diagnosis n (%)					
Unsatisfied	8 (44.4)	6 (40.0)	3 (27.3)	6 (37.5)	23 (38.3)
Satisfied	10 (55.6)	9 (60.0)	8 (72.7)	10 (62.5)	37 (61.7)
Wait time: 1st Visit – Diagnosis (days)					
Median	67.5	91.0	105.0	84.0	84.0
Range	12–723	0–851	14–897	5–642	0–897

Patients’ perceptions of satisfaction with wait times were influenced by three interrelated dimensions: the interpersonal skills of treating physicians, coordination of care across providers, and timeliness of care. There may be overlap between individual items, with many relating to more than one dimension.

### Interpersonal skills

The interpersonal skills of treating physicians relates to the physician-patient relationship. A patient may see multiple physicians as they progress from the investigation of symptoms to treatment. A particularly negative or positive interaction with a single physician may influence their perception of wait time-related satisfaction across multiple providers. Interpersonal skills of treating physicians include expressions of empathy and concern, quality of information exchanges, and accountability for errors.

#### Expression of empathy and concern

Patients expected their physician to be empathetic to their physical and emotional discomfort. They also expected their physicians to speak and act in a manner that expresses concern for the patient. For example, a breast cancer patient whose procedures and test results had been delayed expressed dissatisfaction surrounding the fact that she did not feel her physician cared about her. She lamented: “I feel that I fell through the cracks of the system and… it was like people were thinking, you know, ‘who really cares?’ And I think it’s an awful thing to have that feeling as a patient but that’s the way I was feeling.” {103}. Similarly, while patients realize that physicians may often give bad news, patients nonetheless expect physicians to be sensitive to their feelings. A man who had been matter-of-factly diagnosed with prostate cancer expressed his anger and disappointment with his physician’s apparent lack of concern for his feelings:

He said that 5 out of the 10 samples that they took, that 5 were positive, so he said that means cancer. Now he said, “Is there any questions?” And with that I felt like just plowing him with my fist, (mocking) “now is there any questions?”… [I said] …“You just stuck a knife in me now … you knows I got lots of questions but I can’t even think of what, one right now” {446}.

#### Quality of information exchange

Patients want physicians to provide them with the information they need to make decisions about their care. Patients also want to feel that their questions and concerns are fully addressed. Moreover, while patients want to feel that appointments and procedures are scheduled with minimal delays, they do not want to feel that their appointment is rushed. For example, one prostate cancer patient who went to great lengths to arrange an appointment with a specialist was disappointed about the limited amount of time he had with the physician:

So he gives me the examination, and I’m asking him some questions and [then] he’s walking through the door. And I said, “I’m not finished yet,” … And he just looked at me, blank, you know. And I said, “I’m sorry,” I said, “but this is too, this is commonplace for you,” I said, “you do this over and over, x number of times a day, …“but this is a new game to me.” “It’s something new,” I said, “and we got questions. We got concerns and we got worries.” … He came back and he sat down. We were only in there for about 15 min, but it was 15 min that I wanted not only the 6 or 7 min that he wanted … and he answered all my questions, he put my mind at ease {449}.

Similarly, another prostate cancer patient complained about the lack of information he was provided about treatment options by the physician and the necessity of having to research the options himself:

The only thing that I’m not happy with is the, is the lack of communication you know, between the specialist and the patient … having to choose yourself your own treatment. I can’t imagine somebody who … didn’t get through high school, trying to wade through all this information … and figure out your own treatment {447}.

#### Accountability for errors

While many patients were willing to accept that errors may have been made in their diagnosis and treatment, patients expected their physicians to be accountable for mistakes and delays and apologize. For example, a lung cancer patient experienced delays because he received contradictory advice about treatment options and his test results were misplaced. While he understood that mistakes could happen, he was disappointed that no one on his care team would acknowledge the errors and delays: “If they had sent me a letter and said they were sorry for the delays … and I realize things don’t always go smoothly, and mistakes were made. But, I guess, they weren’t going to admit that …” {334}. Similarly a patient with breast cancer whose portacath was incorrectly inserted was angry that her physician would not admit the mistake: “… why in God’s name he didn’t call and say, ‘I’ve made a mistake, you got to come in, we need to fix that port?’ … I understand people make mistakes.” {103}. Accountability for errors was related to patients’ perceptions of a physician expressing feelings of empathy and concern, but was specific to errors or delays. Patients whose physicians apologized for delays or errors were more likely to feel that their physicians cared about their well-being.

### Coordination

Coordination refers to the patients’ ability to move through the health care system, from one care provider or sector to another. Patients valued seamless transitions. Coordination included system navigation, scheduling of appointments, and sharing of information between providers.

#### System navigation

Helping patients learn and navigate the cancer care system was seen as a key element to coordinating care. In some instances someone (a physician, nurse navigator, or secretary) would help patients understand which physicians or clinics provided each treatment and aid patients in understanding their progression through a variety of caregivers and settings. More often however, patients were blindly referred from one site to another with little explanation of how the individual parts of the system worked together. For example, a patient with colorectal cancer noted his frustration while trying to arrange different visits and treatments. He felt the coordination of care was poor and contributed to increased wait times. He believed he had to navigate the system himself and received very little assistance with arranging appointments or making sure that needed information was available to the right person when needed. His wife (who interjected during his interview) said:

Or if they couldn’t do it [arrange his appointments], they should have said, ‘well, your husband has cancer and you’ll have to try to make arrangements yourself if you can do it yourself.’ Because I had to go to work and make the arrangements anyway…, you don’t know how stressful it is when you know you have cancer and nobody is bothering to help you {223}.

#### Scheduling of appointments

Patients appreciated staff (often secretaries) that understood a patient’s unique situation and made appointments accordingly, often facilitating earlier access to tests or treatments. Patients’ circumstances included their location, willingness and ability to travel to different clinics, employment status, and preferences for various means of delivering care (regular clinics, travelling clinics, tele-health, or alternate provider, etc.). For example, patients who were willing to travel could choose to go to St. John’s or a regional travel clinic. A colorectal cancer patient who was satisfied with his care recalled that he notified the booking secretary that because he was retired, he would be willing to take a last minute appointment:

So I told [the secretary], “I’m retired, if you can give me time enough to get a shower or whatever I got to do, I can come anytime.” And she said “oh great, because,” she said “we have cancellations every single week.” So within 10 min she called me back and she said “I can get you [an appointment] for next week” {226}.

#### Sharing of information

Regardless of their overall wait times, patients valued physicians who arranged appointments and obtained and relayed test results with minimal delays. Patients noted that delays occurred if they were not told test results or, as in the case of one breast cancer patient, there was confusion over who – the family doctor or the specialist – would follow-up with the patient:

… about 2 weeks after [the biopsy] and I called my family doctor and she said, “I think the results are here, didn’t [the surgeon] call you?” and I said, “No.” And then I learned through a friend of mine that the [surgeon was] on vacation, an extended vacation. He was off for a month or something … [after calling the family doctor again] … so my family doctor then gave me the news… so it seemed to be some confusion over who was [supposed to disclose results] {104}.

### Timeliness of care

Timeliness of care refers to the speed and efficiency with which care is provided, with attempts to avoid or mitigate anticipated delays. Timeliness of care includes physicians’ responsiveness to patients’ symptoms, timely follow-up, arrangements for coverage during known provider absences, and shared sense of urgency. Timeliness of care results from a combination of actions by the treating physicians, “the system” (the various clinics and staff involved in the patients care), and patients themselves.

#### Responsiveness to patients’ symptoms

Some patients noted that their family physicians’ reactions to their symptoms set the tone for how quickly or slowly they were diagnosed. Patients who experienced common symptoms, such as upset stomach, often had to seek care multiple times before they felt their physicians took them seriously. A colorectal cancer patient recalled her repeat visits to the doctor: “But my family doctor had no interest whatsoever in finding out what was wrong with me. He kept telling me to take vitamins upon vitamins upon vitamins…” {219}. Another colorectal cancer patient was told that his symptoms would resolve themselves: “[the doctor said] ‘it’s just a little thing, you know, it will go away.’… and finally in May I said to the family doctor, ‘look, this is not going away, it’s getting worse and I want something done’” {224}.

#### Timely follow-up

Patients also noted that avoidable delays were created if their physicians did not disclose results in a timely manner or follow-up to find out test results if they were not available. A colorectal cancer patient appreciated the willingness of his oncologist to follow up and obtain test results: “He would talk to the radiologist and he would get them, you know, within the week or days, right? You know, he didn’t have to wait 3 months or anything, you know. He always made sure that it was done in a timely manner” {221}.

#### Coverage during known provider absences

Patients understood that their physicians may be away from work for various reasons, including treatment for illness, vacation, or other work-related duties. A breast cancer patient noted that her wait for surgery could have been reduced had her surgeon referred her to another physician while the surgeon was on vacation: “I thought maybe that somebody else could speak to me, or, I didn’t expect her to disregard her vacation, but at least reassure me that you know, hey, somebody can see you, you can have, you can consult with somebody else if you want” {101}. Patients appreciated physicians who made alternative arrangements for their care when a physician knew he or she would be away, particularly if the physician’s absence could potentially result in delays in care. For example, a colorectal cancer patient whose doctor noted that she had a troubling blood test made arrangements for follow-up while the doctor was on vacation. In the interview, the patient said: “[the doctor said] ‘I’m leaving this evening for my vacation …I’ll make an appointment, come in Monday morning, and …I’ll get you to see the other doctor again and a follow-up’” {231}.

#### Shared sense of urgency

A shared sense of urgency refers to the concordance between the patient and their care giver’s perception of the need to move expeditiously. For example, a breast cancer patient who was satisfied with her care noted “…it seemed like it was, everybody was very conscious, that it was, that it needed to be seen to, and I thought it was done in a real timely manner” {116}. Similarly, another satisfied breast cancer patient said “And as soon as they realized it was possibly cancer you can certainly see that I was getting rushed through to get everything done. … I noticed that everyone was trying to get me earlier appointments and taking it very seriously. You could see the difference in people around you, you really could” {111}. The sense of urgency is based on the patient’s perception of their condition, rather than the physician’s, as patients may view their condition as more urgent than their care providers. For example, a breast cancer patient who was unsatisfied with her care felt that her care should have been given higher priority said: “You know, there should be some kind of a fast-tracking …a higher priority, triage it” {107}. Although her testing and diagnosis was provided with minimal delay, she nonetheless felt it could have been provided faster.

## Discussion

Through qualitative interviews, we asked cancer patients to describe their care experiences and explain their wait times-related satisfaction. Consistent with other studies, we found that timeliness of care was only one component of wait-related satisfaction [3–6]. Patients’ perceptions of satisfaction were influenced by three domains which related to their interactions with physicians, the coordination of care, and the timeliness of care. The domains overlap and individual items may relate to more than one domain. For example, a physician’s response to a patient’s presenting symptoms may influence the overall time a patient is waiting to receive a diagnosis (i.e. timeliness of care), as well as the patient’s perception of their physician’s concern about their well-being (i.e. interpersonal skills). Recent studies of prolonged time to cancer diagnosis have also highlighted the interconnectedness of items in these three domains. The researchers found that patients with three or more visits to a general practitioner for unresolved symptoms were more likely to experience a pre-diagnostic delay; these patients were also more likely to report negative experiences related to what we have described as interpersonal care (e.g. “information given by general practitioner”), and coordination (e.g. “cancer care integration”) [13].

Researchers from the UK suggest that patient satisfaction assesses whether patients’ personal experiences with the health system measure up to their expectations; that is, satisfaction measures the agreement between expectations and experiences [14]. The findings from our study elucidate patients expectations related to wait times. While intuitively, timeliness may appear to be the most influential determinant of patient satisfaction, the study highlights the importance of the nature of the patient-provider interactions and coordination of care. Other studies have similarly highlighted the importance of communication between the health professionals and the patient in relation to patient satisfaction in cancer care [15, 16] and to timeliness of cancer diagnosis [13].

Our findings also highlight the need to develop valid measures of wait-related satisfaction. Studies to date have relied on single item questions (usually involving a Likert scale) to capture patients’ satisfaction with wait times [2–5, 13]. While a number of patient satisfaction questionnaires have been developed for cancer care, we did not find any instrument that examined satisfaction as patients moved through the health care system from the investigation of symptoms, to diagnosis and treatment. For example, there are a number of validated instruments, which separately measure physician-patient interactions, coordination of care, and timeliness. However, these existing instruments usually refer to a single physician visit or visits at a single site (such as a cancer clinic) [8, 17]. The current study addresses physician-patient interactions, coordination of care, and timeliness as patients move through different sectors of the health care system, from primary care (where patients may present with symptoms and ultimately return for post-treatment monitoring) to specialist and hospital-based care (for diagnostic tests, treatment, and follow-up).

These existing instruments include many of the individual items identified by cancer patients in the study. For example, the quality of communication and physician’s engagement are included in questionnaires examining the physician-patient relationship [18–20]. Similarly, communication between care providers and integration of services are captured in instruments measuring coordination of cancer care [21–23] and timeliness of care [24, 25]. The agreement between the domains and individual items in our study and existing instruments strengthens the credibility of the study findings. Moreover, the current study illustrates how these individual constructs relate to patient satisfaction with wait times.

Findings from this study provide a conceptual basis for the creation of a scale to assess wait-related satisfaction. Future research is needed develop and validate an instrument that can be used to compare wait-related satisfaction across different cancer types and between jurisdictions. By identifying the dimensions of wait-related satisfaction, we also highlight possible strategies to improve patients’ satisfaction with wait times for care. Improving physician-patient interactions and coordination of care, as well as reducing wait times for tests and treatment, will all contribute to greater wait related-satisfaction for cancer care.

### Limitations

The data are self-reported and subject to patient recall. The retrospective design of the study, with diagnoses and prognoses known, may have influenced perceptions of waits. More research is needed to examine patients with other cancers, with multiple cancers, and residing in other provinces. Our study interviewed individuals who had a confirmed diagnosis. Further research is needed to examine the experiences of individuals who were ultimately not diagnosed with cancer. Their experiences will provide a more complete description of patients’ experiences of symptom investigation. Individuals who do not receive cancer diagnoses may view their experiences differently than those who receive a cancer diagnosis if the diagnosis provides a sense of purpose or explanation to the patients’ experience.

## Conclusion

Using qualitative interviews with cancer patients, we described the dimensions of wait-related satisfaction: physicians’ interpersonal skills, coordination of care, and timeliness of care. These interrelated and often overlapping categories describe the aspects of care that contribute to patients’ satisfaction or dissatisfaction with wait-related experiences as patients move through the health care system from the onset of their symptoms to their cancer treatment. These findings provide the conceptual basis for the development of validated instruments to measure wait time-related patient satisfaction.

## Abbreviations

NL: Newfoundland and Labrador
